# Lived Experiences of Deathcare Workers in Managing Infectious Dead Bodies

**DOI:** 10.1002/puh2.70205

**Published:** 2026-02-27

**Authors:** Nkosi Nkosi Botha, Cynthia Esinam Segbedzi, Victor Kwasi Dumahasi, Ruby Victoria Kodom, Mary Aku Ogum, Samuel Maneen, Ivy Selorm Tsedze, Lucy Adjanor Akoto, Edward Wilson Ansah

**Affiliations:** ^1^ Department of Health Physical Education and Recreation University of Cape Coast Cape Coast Ghana; ^2^ Air Force Medical Centre Ghana Armed Forces Medical Services Air Force Base Takoradi Ghana; ^3^ Institute of Environmental and Sanitation Studies, Environmental Science, College of Basic and Applied Sciences University of Ghana Accra Ghana; ^4^ Department of Health Services Management/Distance Education University of Ghana Accra Ghana; ^5^ Department of Adult Health School of Nursing and Midwifery University of Cape Coast Cape Coast Ghana

**Keywords:** Ghana, infection prevention and control, manipulation of dead bodies, occupational health, psychosocial hazards, stigma

## Abstract

**Introduction:**

Deathcare workers in resource‐limited countries are chronically exposed to infectious diseases, mainly due to a lack of effective safety controls, inadequate resources, poor training and laxity on the part of industry managers. However, there is limited evidence from these countries, and very little is known about how deathcare workers manage infectious dead bodies. The aim of this study is to explore the experiences of deathcare workers involved in the management of infectious dead bodies in Ghana.

**Methods:**

Using purposive sampling, data were collected from 32 deathcare workers using 11‐item semi‐structured in‐person interview guide. An observation checklist was also used to gather additional data on on‐site safety practices. Thematic analysis was conducted using the realistic phenomenological approach.

**Results:**

We found that there were no clearly defined safety control systems (engineering, administrative and personal protective equipment supply and use) in place to protect workers against infectious diseases. For example, there were no separate storage facilities for infectious dead bodies. The workers were also exposed to various types of psychosocial hazards, such as heavy workload and dirty and disorganised work environments, which could affect their self‐esteem, life satisfaction and coping skills. Additionally, these workers reported feeling humiliated, receiving poor remuneration and lacking opportunity for career progression.

**Conclusions:**

The deathcare workers in Ghana were not adequately prepared to handle infectious dead bodies, which put them at risk for infection and increased the psychosocial hazards at work. These workers may resort to unhealthy coping strategies, which require urgent attention. Future research should use qualitative approaches to investigate the working relationships between deathcare workers and their managers.

## Graphical abstract

Deathcare workers in this study lack the necessary resources, training, and skills to safely manage infectious dead bodies. Consequently, the workers are exposed to infection and various types of psychosocial hazards, including heavy workload and unsanitary work environments. These factors may affect their self‐esteem, life satisfaction, health and overall well‐being. These workers may resort to unhealthy coping strategies, which require urgent attention.

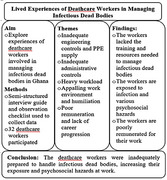

## 1 Introduction

The threat of infection among mortuary attendants and funeral directors; here referred to as deathcare workers, remains a significant work‐related hazard globally [1, 2, 3, 4, 5, 6, 7, 8, 9, 10]. The daily work routines of these workers include carrying dead bodies, cleaning and embalming dead bodies, as well as cleaning equipment and tools used for such activities [1, 2, 3, 4, 5]. These activities bring these workers into direct and regular contact with dead bodies, exposing them to the risk of infection. For example, they are constantly at risk of being splashed with body fluids from the orifices (mouth, nose, eyes etc.) of the dead bodies, which can enter their body through their mouth, nose, eyes and parts of the body [2, 3, 4, 5, 6]. Studies suggest that the risk of infection is particularly concerning when performing post‐mortem or embalmment procedures and also when handling decomposing, malodorous, mutilated and disfigured dead bodies [3, 4, 5, 6, 7]. Typical are dead bodies that have been exhumed and accident cases. Although the risk of infection from dead bodies is widely reported [1, 2, 3], several other studies [1, 2, 3, 4, 5] suggest that all dead bodies are potentially infectious and that there must be effective and efficient safety controls in place to protect the workers.

However, deathcare workers have historically faced negative occupational identity as society perceives their work as ‘dirty’ and prone to infection [1, 2, 3, 4, 5, 6, 7, 8, 9, 10]. This negative characterisation has led to rejection, stigmatisation and repugnance towards these workers [1, 2, 3, 4, 5, 6, 7, 8, 9, 10]. These experiences were widely reported among globally during the recent global outbreak of the COVID‐19 pandemic [2, 11, 12, 13, 14, 15, 16, 17]. Notably, deathcare workers in the global south were disproportionately impacted by the global pandemic due to inadequate protection while managing large numbers of dead bodies [4, 5, 18, 19, 20].

Moreover, the risk of infection and the associated psychological burden among deathcare workers in Ghana and other sub‐Saharan African (SSA) countries continues to increase due to the outbreak of new and re‐emerging infectious diseases, such as COVID‐19, Ebola, Marburg, Mpox, Lassa, Dengue and Cholera [21, 22, 23, 24, 25]. Unfortunately, most deathcare facilities in Ghana and other SSA countries lack adequate facilities and logistics for effective infection prevention and control (IPC) [16, 18, 19]. Additionally, deathcare workers in these countries are inadequately trained, poorly resourced, poorly monitored and supervised and poorly remunerated. There is also limited evidence on how deathcare workers in SSA manage infectious dead bodies.

In Ghana, studies exploring deathcare services are scarce and still emerging [16, 17, 18, 19]. For example, Botha et al. [16] and Dartey et al. [19] reported that mortuary attendants were not formally educated and mostly learned the job through apprenticeship. These studies suggest that deathcare workers in Ghana do not adhere to the universal standard precautions for handling dead bodies due to poor educational backgrounds, inadequate training and lack of resources [16, 18, 19]. Additionally, these workers were found to be frequently exposed to biological, ergonomic and psychological hazards at work [16, 17]. For instance, they experience needle stick injuries [18], workplace violence and loud noises and have irregular work schedules [16], resulting in sleep disorders and self‐medication [16, 17, 19]. Therefore, this study aims to explore the experiences of deathcare workers involved in the management of infectious dead bodies in Ghana.

## 2 Materials and Methods

### 2.1 Research Design

We conducted this study using the realistic phenomenological (RP) design, incorporating observation, to explore the experiences of the deathcare workers in managing infectious dead bodies in Ghana. This design was chosen because it allows for the integration of both interview and observational data [26, 27]. To ensure accuracy and integrity of our data, we utilised various qualitative approaches, including variation, description, introspection and bracketing, to report on the experiences of these workers. Through descriptive analysis, we immersed ourselves in the data to gain a deeper understanding of workers’ lived experiences [26, 27]. We also used introspection to gain a broader perspective on these lived experiences [26, 27] and employed bracketing to minimise our own biases assumptions from influencing the study findings [26, 27]. Additionally, the variation method was employed to uncover different dimensions of the data [26, 27].

### 2.2 Study Area

The study was conducted in three deathcare facilities, purposefully selected from the Central and Western North regions of Ghana. The first deathcare facility is located in the Western North Region and has a capacity of 120 dead bodies [28]. It is the main public deathcare facility located in the Aowin Municipality, near Ivory Coast, which has had a history of Lassa fever. This facility receives bodies from various locations, including Ivory Coast, which is why we chose to include it in our study. The second facility is a privately owned deathcare provider, also located in the Aowin Municipality, and has 14 workers [29]. Although this facility does not have a mortuary, it receives and transports bodies from the morgue to designated sites or its premises. Here, the bodies are cleaned, dressed and prepared for public viewing and other rituals. We were interested in this facility due to the large number of workers, specifically funeral directors that work with it. The third facility, also privately owned, is located in the Komenda–Edina–Eguafo–Abrem (KEEA) Municipality in the Central Region [30]. This facility was chosen because it is the largest private deathcare provider in the municipality with a holding capacity of 150 dead bodies. It offers both storage and funeral services to communities near and far.

### 2.3 Sampling and Ethical Considerations

From a total population of 38 deathcare workers at these three facilities, we used purposive sampling method to select 32 (84%) workers (first facility—6, second—14 and third—12) who were available, willing and gave informed consent to participate in the study. Given that the study population was relatively small (38), we were interested in using every deathcare worker [31], as long as the worker was available and consented to participate in the study. Therefore, we adopted the total population sampling technique by Nikolopoulou [32], which was useful in attaining saturation. We believed that a sample of 32 deathcare workers would help establish credibility and trustworthiness of our findings [31]. Six of the workers (16%) declined to give informed consent and did not participate in the study. Out of the 32 participants, 21 (65.6%) were males.

The study protocols were submitted to and approved by the Institutional Review Board of the 37 Military Hospital, Accra (37MH‐IRB/PhD/IPN/817/23). Both the deathcare workers and their managers gave verbal or written informed consent to participate in the study. This was after the objective of the study was explained to them. Recruitment and data collection commenced on 10 February 2024 and ended on 23 August 2024. Three deathcare managers (supervisors) from each of the selected facilities were included in the study to obtain additional data to confirm the reports from the deathcare workers (triangulation).

### 2.4 Data Collection

A semi‐structured interview guide was purposely developed and used for face‐to‐face worker interviews. Additionally, workplace hazards observation checklists were developed to facilitate collecting additional data for the study [16, 17, 18, 19]. The 11‐item interview guide was divided into two parts: Part I included demographic information, years of educational and work experience of the workers, and Part II focused on specific hazards of working with infectious disease dead bodies, as well as on‐site psychosocial hazards experienced by the workers. Sample questions included: ‘How were you prepared to handle infectious bodies? What measures are in place to protect you against infectious diseases? What psychosocial hazards do you experience during work?’ Interviews lasted between 30 and 45 min and were conducted at work during break times, where we took audio recordings and field notes of the interactions.

We also used an observational checklist to explore on‐site safety practices of the workers regarding handling of infectious disease dead bodies and the state of the internal and external work environments. Some items included ‘personal hygiene, handling of dead bodies, and availability and use of personal protective equipment (PPE), and availability of water’. The observations were carried out during peak work hours work (Fridays and Saturdays) when workers were busiest. Please refer to ‘Appendix A’ for details on the instruments.

### 2.5 Pre‐Testing

The instruments were pre‐tested using a sample of 4 mortuary attendants and 3 funeral directors. Audio recordings were transcribed and reconciled with field notes, which were analysed using the RP approach [30, 31, 32, 33, 34, 35, 36, 37, 38, 39]. Two experts assessed the instruments for their content validity and reliability.

### 2.6 Analysis

A four‐step RP data analytic approach was used. It commenced with the data collection [30, 31, 32, 33, 34, 35, 36, 37, 38, 39]. First step: The audio recordings were transcribed verbatim and compared with the field notes and observation reports. To uphold worker confidentiality, all names mentioned during the interview were anonymised (see Tables 1 and 2). Key words and phrases that aligned with the objectives of the study were identified from the audio tapes, field notes and observation reports. Using a data coding book, initial codes were then identified and developed by reading the transcripts repeatedly until data saturation was attained [30, 31, 32, 33, 34, 35, 36, 37, 38, 39]. Similar codes were developed into themes and screened for cohesion. The emerging themes were modelled and rephrased to ensure comprehension and consistency [30, 31, 32, 33, 34, 35, 36, 37, 38, 39]. Thematic analysis was conducted using NVivo version 14. Guided by the purpose of the study, a comprehensive summary of the analysis was developed to affirm the fundamental structures of the phenomenon under study.

**TABLE 1:** Demographic characteristics of deathcare workers.

Variable	Frequency
**Gender**	
Male	21
Female	11
**Age**	
20–30	2
31–40	18
41–50	10
51–60	2
**Level of formal education**	
Primary school	5
Middle school/JHS	6
Secondary school	19
Diploma	2
**Years of experience**	
2–5 years	10
6–10 years	18
11 years and above	4
**Work setup**	
Public	6
Private	26

**TABLE 2:** Demographic characteristics of deathcare managers.

Ser	Pseudonym	Sex	Age	Level of formal education	Work experience (years)
1	FDC53PMGRCR	M	53	SHS	12
2	MDC46GMGRWNR	M	46	Degree	7
3	FDC47PMGRWNR	M	47	SHS	8

### 2.7 Data Verification, Validation and Reflexivity

To ensure trustworthiness and rigour, we contacted some participants to verify and validate some significant observations made in the dataset. Throughout the study, we kept a reflexive journal to document the detailed backgrounds of all investigators. We proactively discussed and critically examined our personal fears, emotions, reservations, stereotypes and misconceptions about the deathcare industry to mitigate potential biases that could influence the study's conduct. We also addressed the potential impact of our personal characteristics on the quality of interviews, observations, data transcription, interpretation and conclusions during peer debriefing. This helped as to stay focused on the direction of the data rather than our personal views.

## 3 Results

Two themes and five sub‐themes emerged from the data. Refer to Table 3 for a summary of themes, sub‐themes and codes that emerged from the data.

**TABLE 3:** Summary of themes, sub‐themes and codes that emerged.

Themes	Sub‐themes	Emerging codes
1. Weak engineering and administrative controls	Inadequate engineering controls and PPE supply	Poorly designed storage facilities, no provision for infectious dead bodies, inadequate handwashing facilities, lack of personal hygiene facilities, no proper landscaping, inadequate and poor‐quality hand gloves and nose masks, inadequate coveralls and safety boots
	II Inadequate administrative controls in handling infectious dead bodies	Lack of training, lack of supportive monitoring and supervision, no staff meetings with the manager, not involved in procurement decisions
2. Psychosocial hazards	Heavy workload	Too much work, inadequate number of personnel, lack of annual leave, fatigue and exhaustion, frustration and lack of interest, falls and slips, headache and dizziness
	II Appalling work environment and humiliation	Unpleasant smell of the environment, unkempt workspace, wall paintings were peeling, non‐functional handwashing sinks, faulty electrical fittings, poor drainage systems, decomposing bodies, loosely hanging electrical wires, dirty and non‐functional sinks, poor lighting system, poor ventilation, insulted and disrespected by managers, neglect by managers, other staff of the hospital avoid our company, irregular water supply
	III Poor remuneration and lack of career progression	Low salaries, inability to support family, not considered for study leave, lack of medical screening and care, no welfare scheme, no social support systems and lack of promotion

Abbreviation: PPE, personal protective equipment.

### 3.1 Theme One—Weak Engineering and Administrative Controls

**FIGURE 1:**
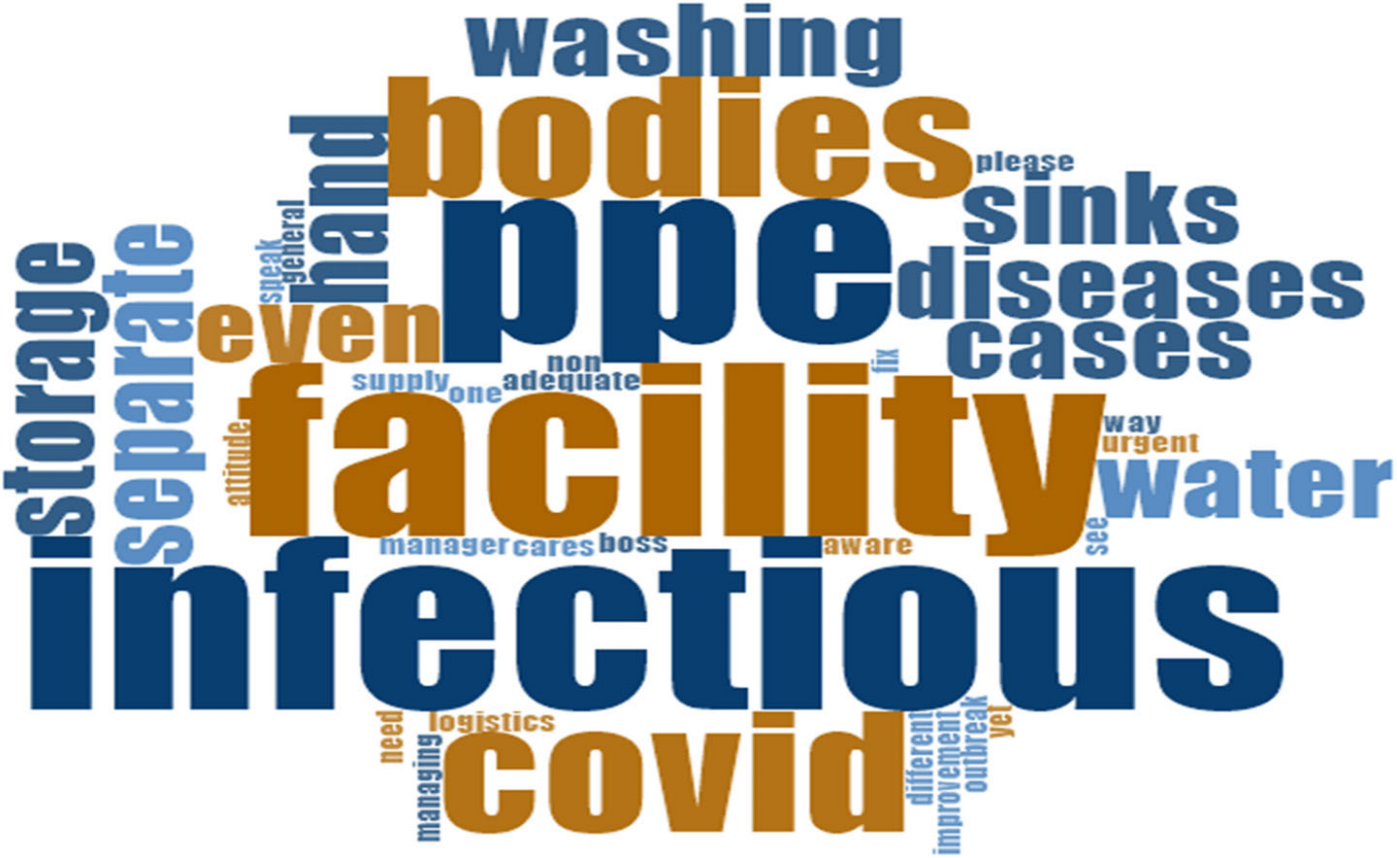
Words used by participants during interview on facilities for infectious bodies. *Source:* Word Cloud generated from the Qualitative Computerised Data Software, NVivo version 14.

This theme produced two sub‐themes: (i) inadequate engineering controls and PPE supply and (ii) inadequate administrative controls in handling infectious disease dead bodies (Figure 1).

#### 3.1.1 Sub‐Theme One: Inadequate Engineering Controls and Supply of PPE

It was found that deathcare facilities lacked the necessary engineering controls for safe management of infectious deceased bodies. For example, there were no specialised storage facilities for dead body preservation. Furthermore, hand and personal hygiene facilities, as well as other logistics like PPE, were inadequate for safe handling infectious disease dead bodies. In response, a manager, MDC46GMGRWNR, remarked: *‘*… of course, we do not have separate facilities for storing such cases [infectious bodies]. However, whenever such cases are received, a portion of the cold room is dedicated to them’. (7 years as a deathcare worker). This practice is problematic due to the high likelihood of cross‐contamination with non‐infectious deceased bodies.

Moreover, a single cholera case, for example, constitutes an outbreak and requires strict adherence to IPC [36, 37, 38]. A worker, SP84M60CR, lamented: ‘… we do not even have adequate PPE and supporting logistics for non‐infectious cases, let alone infectious ones. By the way, who cares? There was no improvement in our PPE supply, even during the COVID‐19 outbreak. …’ (6 years, 4 months as a deathcare worker). Meanwhile, there was no significant difference between public and private deathcare facilities regarding the availability and adequacy of engineering controls for managing infectious deceased bodies.

#### 3.1.2 Sub‐Theme Two: Inadequate Administrative Controls in Handling Infectious Disease Dead Bodies

**FIGURE 2:**
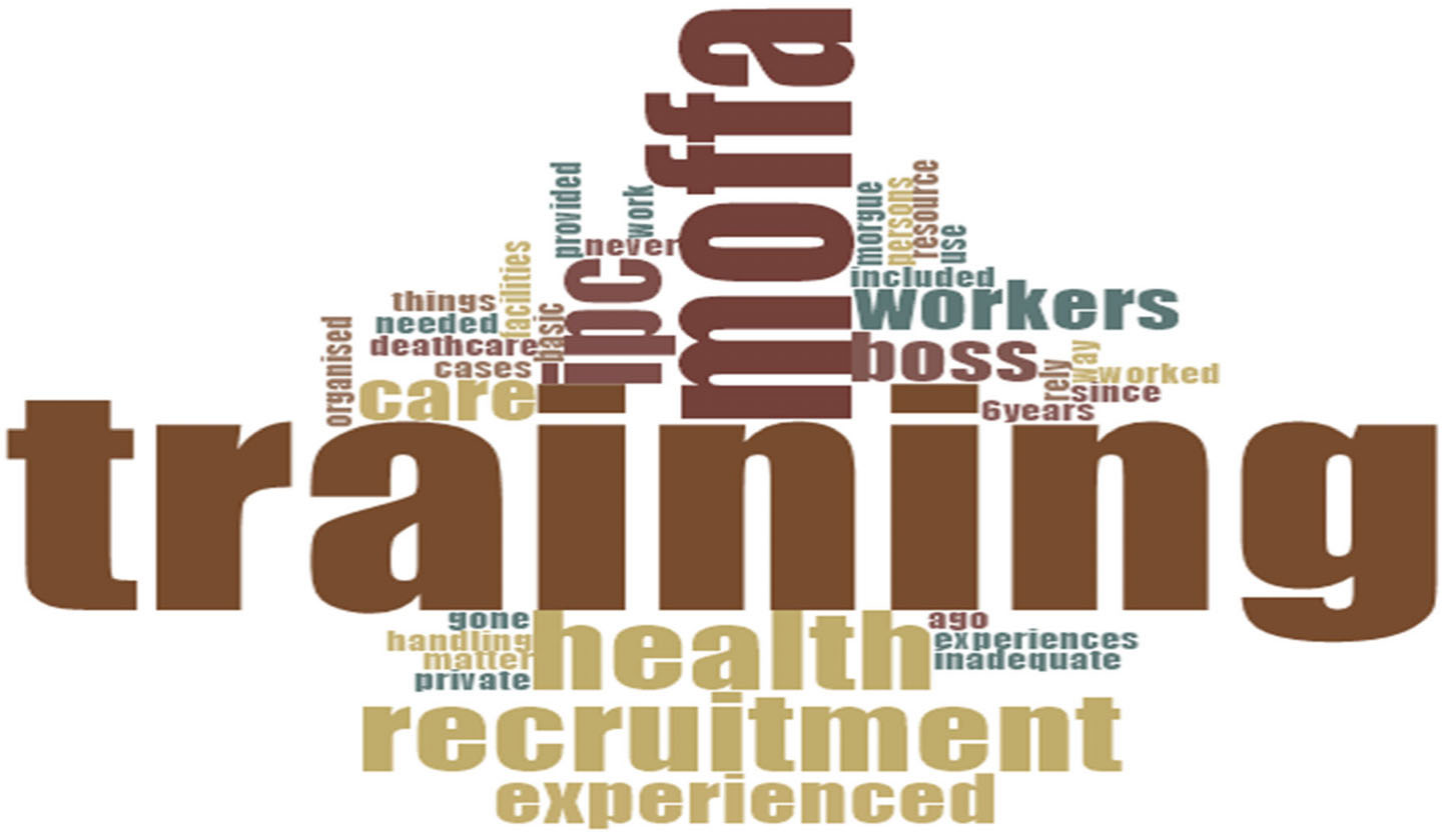
Words used by participants during interview on trainin. *Source:* Word Cloud generated from the Qualitative Computerised Data Software, NVivo version 14.

We also found that the administrative measures in place were inadequate to guarantee total protection for workers against infections from such dead bodies. For instance, workers were not adequately trained to safely manage infectious deceased individuals (Figure 2). Apart from a single IPC training organised by the Mortuaries and Funeral Homes Agency in collaboration with the Mortuary Workers’ Association—Ghana (MoWAG) for selected mortuary workers from public facilities, most workers had not received any formal training since recruitment. A worker, SHS33F60CR, cried: ‘I have never been trained since being recruited 6 years ago. Why has Mortuaries and Funeral Facilities Agency [MoFFA] excluded us, private facility workers, from IPC training? Are we not also deathcare workers?’ (6.5 years as a deathcare worker). This is unfortunate, as the situation can expose workers to unsafe practices, resulting in infection and other health issues.

The workers’ practices exposed gaps in administrative controls (handwashing standards) and PPE use, confirming a lack of requisite knowledge and skills in IPC. Some workers believed their extensive experience as deathcare professionals qualified them to safely handle any type of deceased individual, even without formal training. For example, worker SHS14M01CR, with 10 years and 2 months of service, remarked: *‘*What is the use of training when the very basic things we needed for work are not provided or are inadequate?’ This perspective may stem from personal and organisational factors, including limited educational backgrounds, negative perceptions of deceased individuals, insufficient training and a lack of supportive monitoring and supervision.

### 3.2 Theme Two: Psychosocial Hazards of Work

**FIGURE 3:**
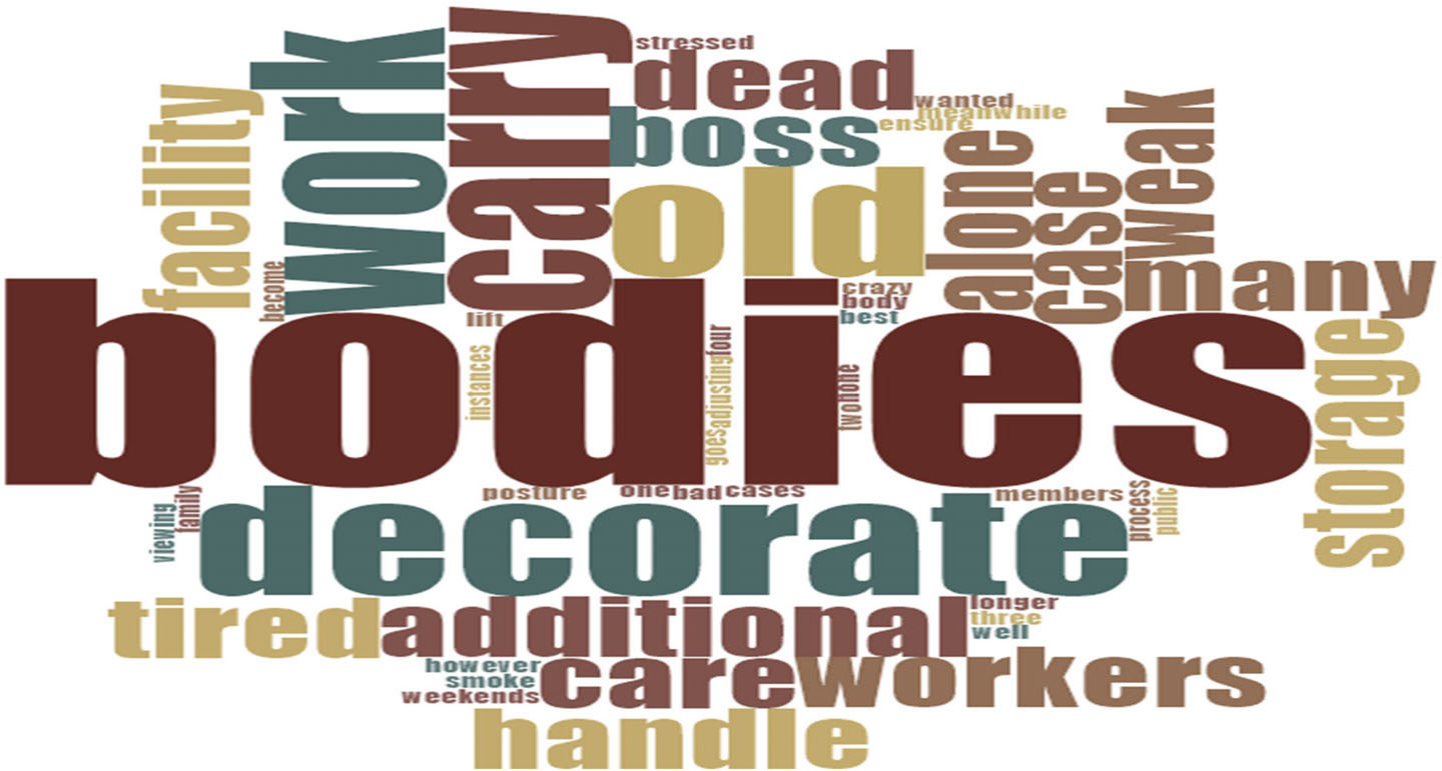
Words used by participants during interview on workload. *Source:* Word Cloud generated from the Qualitative Computerised Data Software, NVivo version 14.

**FIGURE 4:**
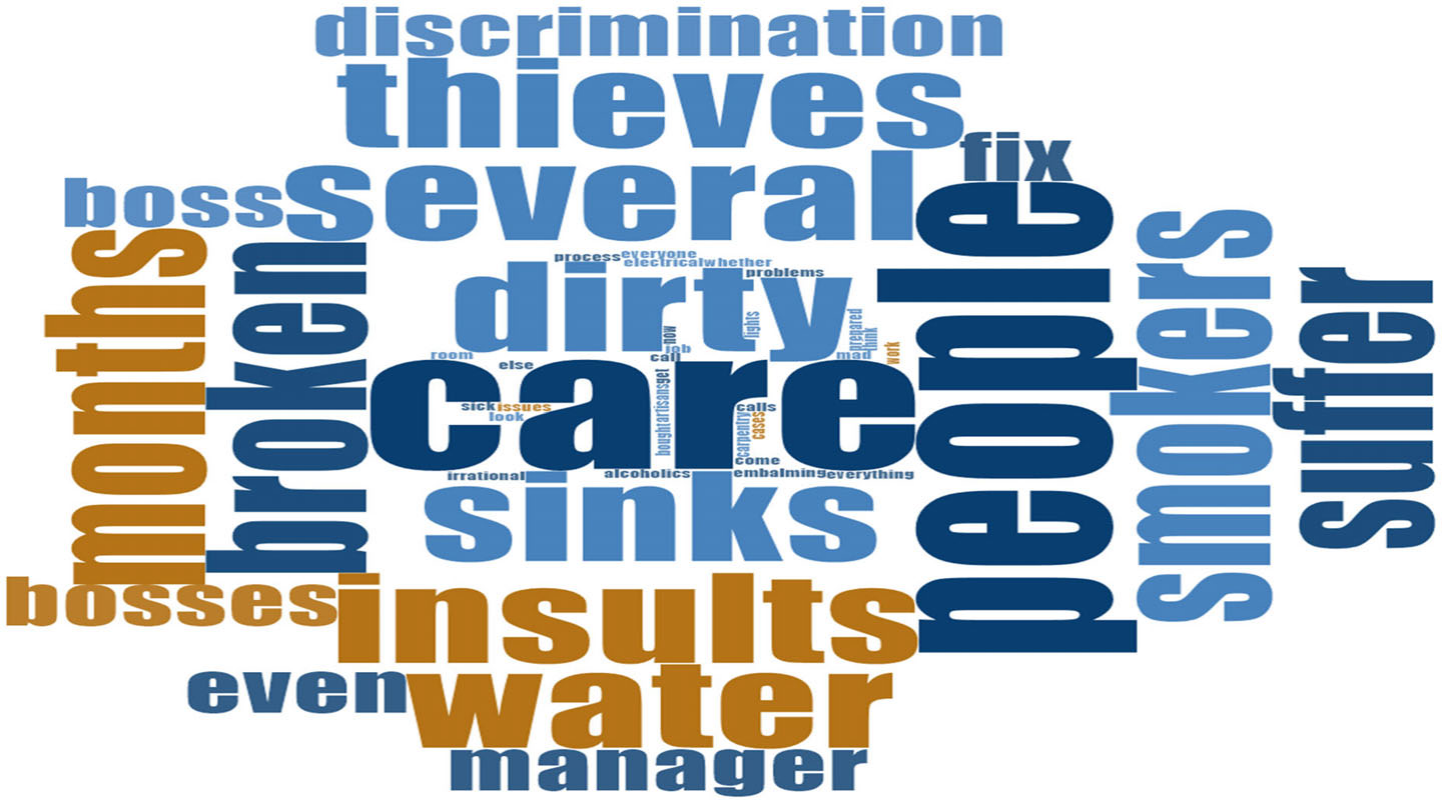
Words used by participants during interview on work environment and humiliation. *Source:* Word Cloud generated from the Qualitative Computerised Data Software, NVivo version 14.

**FIGURE 5:**
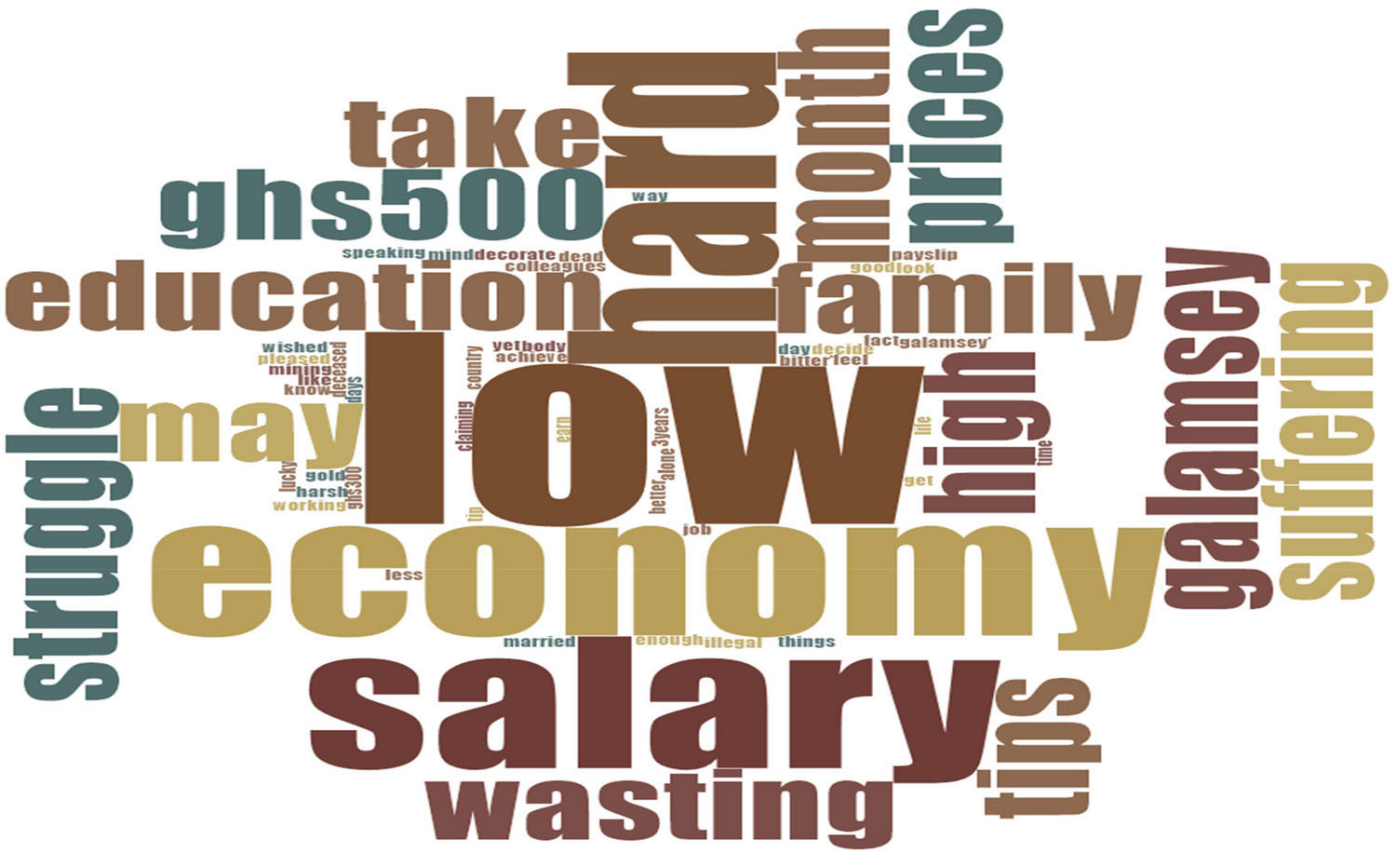
Words used by participants during interview on remuneration and carrier progression. *Source:* Word Cloud generated from the Qualitative Computerised Data Software, NVivo version 14.

This theme produced three sub‐themes: (i) heavy workload, (ii) appalling work environment and humiliation and (iii) poor remuneration and lack of career progression (Figures 3, 4, 5).

#### 3.2.1 Sub‐Theme One: Heavy Workload

The number of deathcare workers in both private and public facilities is inadequate for handling deceased bodies, which could compromise adherence to safety standards. Moreover, some employees had worked for years without leave, resulting in stress, burnout and loss of concentration. One worker lamented: ‘There were instances where only two of us had to prepare the dead body for public viewing, and you can go crazy [become stressed out]’. (7 years, 3 months as a deathcare worker). Another worker, SHS44M70CR, complained: ‘We told our boss that we needed additional help, but the feedback was insulting. He said we could resign if we could no longer do the job’. (7 years as a deathcare worker). This reflects cheap labour, which is resulting in a poor worker–employer relationship, severely undermining the organisation's work climate and safety culture. When contacted about this, a deathcare manager, MDC46GMGRWNR, unfortunately remarked: ‘Yes, additional staff are needed at the morgue, but we also need Medical Officers and Nurses’. (7 years as a deathcare worker).

#### 3.2.2 Sub‐Theme Two: Appalling Work Environment and Humiliation

We also found some common psychological safety hazards, including the generally poor state of deathcare facilities and the stigma attached to the deathcare work. Most facilities were malodorous, had irregular water supplies and lacked proper waste disposal systems. The work environment was generally untherapeutic and unconducive to effective and efficient work. A worker, SHS04M50WNR, cried out: ‘… the sinks have been broken for several months now, and our boss [manager] does not care. It is difficult to find artisans to fix our problems’. (5 years as a deathcare worker). SP54M01WNR, a deathcare worker of 10 years, lamented: ‘Everyone insults us because of our job. They call us “weed smokers”, “alcoholics”, “mad people”, “irrational”, “dirty” and everything else you can think of’. This finding reflects weaknesses in the engineering and administrative controls and a neglect at the facilities under study. Meanwhile, Ghana's Labour Law and Sustainable Development Goal 8 advocate for decent and supportive work environments for all working people.

#### 3.2.3 Sub‐Theme Three: Poor Remuneration and Lack of Career Progression

The study also revealed a lack of clear career progression opportunities for these workers. Most also felt disappointed and demoralised by their poor remuneration and unclear career paths. One worker, SHS73M70CR, expressed frustration, stating: ‘This is my payslip… how do I take care of my family? I would be better off doing “galamsey” [illegal gold mining]’. (7 years, 3 months as a deathcare worker). The poor conditions of service for these workers may stem from their limited educational backgrounds and inability to pursue further education for improved employment opportunities. Another worker, SHS24M30WNR, criticised: ‘After 3 years on this job, I earn less than GHS500.00 [$32] a month’. (3 years as a deathcare worker). In response, a manager, FDC53PMGRCR, commented: ‘I promise to increase their monthly salaries when things improve, but for now, that is all I can afford’. (12 years as a deathcare worker).

## 4 Discussion

The experiences of deathcare workers in managing infectious deceased bodies and the associated psychosocial hazards of their work were explored. Engineering and administrative safety controls at these deathcare facilities were suboptimal. Furthermore, the supply, availability and use of Personal Protective Equipment (PPE) were highly inadequate to guarantee optimal protection for workers caring for infectious deceased bodies. Consequently, workers were ill‐prepared to safely manage infectious deceased bodies, leading to numerous physical and psychosocial hazards at work.

The Occupational Health and Safety and IPC Policy Guidelines of the Ministry of Health, Ghana [40, 41], mandate managers of deathcare facilities to implement adequate engineering controls to prevent infections at mortuaries. Contrary to these guidelines and the decent work agenda proposed by the International Labour Organisation (ILO) [26], this study revealed that deathcare facilities lacked appropriate safety infrastructure for the safe management of infectious deceased bodies. These facilities had no specially designed storage for infectious deceased bodies, and personal hygiene amenities were insufficient for their safe handling. This aligns with previous studies [15, 19, 20] reporting similar findings in SSA. Meanwhile, the World Health Organization (WHO) [25] called for adequate safety controls, including a sufficient supply of PPE, to protect deathcare workers against the COVID‐19 outbreak in Africa. The current study's findings suggest that deathcare workers in Ghana are at high risk of contracting infections from deceased individuals, as there were no dedicated facilities for safe management of infectious bodies, and personal protective measures were highly inadequate.

Training is an effective administrative control measure and a prerequisite for safe management of infectious deceased individuals. Occupational Health and Safety and IPC Policy Guidelines mandate facility managers to provide workers with regular training in IPC and safe management of infectious deceased individuals. However, consistent with previous studies in SSA [15, 16, 20] and Latin America [1, 2], we found that workers were not adequately trained in safe management of infectious deceased bodies. These workers relied on personal experience and exercised broad discretion in managing such dead bodies. Multiple factors might explain the current study's situation, with a lack of management commitment being a potential cause. These factors can be categorised personal or institutional. Personal factors include an individual's attitude towards safety, level of formal education, awareness and knowledge of occupational hazards, safety participation and awareness and knowledge of deathcare industry policies and operational standards [40, 41, 42]. Institutional factors, such as resources, logistics, hygiene, sanitary facilities, workload, training, orientation, supportive monitoring, supervision, medical screening and overall workplace safety culture, align with the collective responsibilities of workers and managers. These responsibilities are outlined in Ghana's deathcare regulations, including the IPC policy, Occupational Health and Safety policy and Labour Law. These factors are universally relevant to the deathcare industry.

Workforce burnout and exhaustion have been widely reported [1, 2, 12, 20, 21] as common health and safety consequences of heavy workloads among deathcare workers in Africa. Ghana's Labour Law [43, 44] and the decent work agenda [26] provide for workloads that commensurate with the strengths and abilities of employees. Contrary to this, it was found that the number of workers at deathcare facilities was inadequate to safely manage dead bodies. Moreover, weak engineering control measures, such as the equipment and facilities needed to perform certain tasks, were lacking. This affirms findings from previous studies in Africa [15, 16, 20, 22] and elsewhere [1, 2]. Furthermore, we found that deathcare managers were indifferent to this precarious situation, perceiving the heavy workload as an integral part of deathcare work that employees must be prepared to endure. This constitutes cheap labour that abuses workers both physically and psychologically, requiring urgent redress. The implications are that employees work long hours without adequate rest, become exhausted and lose concentration needed for effective and efficient work, including self‐protection.

The physical work environment of a deathcare facility can significantly influence employee morale and public perception of the industry, including its workers [3, 11, 45]. Ghanaian Labour Law and ILO standards require deathcare managers to ensure a decent work environment, free from elements that threaten the psycho‐physiological health, safety and well‐being of employees. Specifically, the work environment must be clean, well‐painted, adequately ventilated and equipped with washrooms, rest rooms, furniture, air conditioned and hygiene facilities. Sadly, the work environment of deathcare facilities in the current study does not align with these policies and is precarious and highly exposing the workers. As a result, and consistent with previous studies [15, 16, 20, 21, 22], workers suffer humiliation from the general public, who perceive the deathcare industry as ‘dirty’. Given the generally poor state of psycho‐physical work environments, workers were likely to suffer low self‐esteem, which increases the adoption of maladaptive coping strategies injurious to their health and well‐being.

Remuneration and career progression are two very important prerequisites for a healthy psychosocial work climate and for improving worker morale [26, 46]. Consistent with findings from a previous study in Africa [6], the current study revealed no clear opportunities for personal development for these workers. Additionally, their incomes were inadequate to guarantee better social protection or a comfortable retirement, which also reduced their ability to care for their families. This situation could easily induce workers to engage in corrupt practices, such as charging unapproved fees or extortion while others abuse substances.

## 5 Strengths and Limitations

This study was conducted among two cadres of deathcare workers; mortuary attendants and funeral directors, at three deathcare facilities (public and private) across two regions (Central and Western North) in Ghana. This strategy allowed for data collection from diverse sources, ensuring triangulation and producing rich data that validate our findings. Additionally, pre‐testing the instruments and thorough methodological application provide clear and comprehensive explanations, improving the study's reliability, validity and overall reproducibility. However, sample size of 32 deathcare workers from three facilities and the qualitative approach limit the generalisability of our findings to other deathcare facilities within and outside the Central and Western North regions. Additionally, the findings are specific to the Ghanaian context, suggesting a need for future studies to include other SSA settings. The lead author's background as a health services administrator with oversight of mortuary facilities may have introduced some bias in interpreting the experiences of workers and their managers. Meanwhile, we bracketed ourselves to prevent personal biases and preconceptions about the deathcare industry from unduly influencing the study's findings and conclusions.

## 6 Recommendations for Policy and Future Research Directions

The Ministry of Health, Ghana, and the Mortuaries and Funeral Facilities Agency, in collaboration with deathcare managers and other stakeholders, should develop a policy guideline to regulate worker recruitment within the industry. This would standardise recruitment procedures and ensure engagement of individuals with appropriate academic and professional qualifications. Ultimately, such a policy would improve and rebrand the industry by triggering changes in the general conditions of service for workers. The Ministry of Health, Ghana, the Mortuaries and Funeral Facilities Agency, and deathcare managers must develop a training program to significantly enhance workers’ knowledge and skills in safely managing infectious disease cadavers. Additionally, a deathcare facility assessment scheme is needed for periodic evaluation and monitoring to improve physical work environments. Future research could quantitatively investigate the psycho‐physical safety conditions of public and private deathcare facilities in Ghana. Moreover, future research may quantitatively examine psychosocial hazards and work‐life balance among deathcare workers in Ghana. Additionally, future qualitative research may explore the working relationship between deathcare workers and their managers in Ghana and how that is affecting workers’ health and well‐being.

## 7 Conclusions

The study revealed that deathcare workers in selected facilities across Ghana's Central and Western North regions were inadequately prepared to manage infectious disease cadavers. Specifically, these facilities lacked the necessary infrastructure for safe cadaver management, because the workers had not received training in this area. Additionally, workers were consistently exposed to numerous psychosocial hazards, including heavy workloads, poor working conditions and humiliation. Furthermore, they were poorly compensated and lacked opportunities for career advancement. The facilities’ physical work environments did not conform to the Occupational Health and Safety and IPC Policy Guidelines or the ILO's decent work agenda. These guidelines ensure adequate social protection and respect for human beings, including workers. Workers were likely exposed to high levels of infection, potentially leading to infectious disease outbreaks and public health emergencies. These workers might also adopt unhealthy coping strategies, such as charging unapproved fees, extortion or substance abuse, which could compromise their health, safety, well‐being and the industry's overall image.

## Author Contributions

Nkosi Nkosi Botha, Edward Wilson Ansah, Cynthia Esinam Segbedzi, Victor Kwasi Dumahasi and Ruby Victoria Kodom conceptualised and designed the review protocols. Edward Wilson Ansah, Cynthia Esinam Segbedzi, Victor Kwasi Dumahasi, Ruby Victoria Kodom, Mary Aku Ogum, Samuel Maneen, Ivy Selorm Tsedze, Lucy Adjanor Akoto and Nkosi Nkosi Botha conducted data collection and acquisition. Edward Wilson Ansah, Cynthia Esinam Segbedzi, Victor Kwasi Dumahasi, Ruby Victoria Kodom, Mary Aku Ogum, Samuel Maneen and Nkosi Nkosi Botha carried out extensive data processing and management. Edward Wilson Ansah, Cynthia Esinam Segbedzi, Mary Aku Ogum, Victor Kwasi Dumahasi and Nkosi Nkosi Botha developed the initial manuscript. All authors edited and considerably reviewed the manuscript, proofread for intellectual content and consented to its publication.

## Funding

The authors have nothing to report.

## Ethics Statement

The study protocols were submitted to and approved by the Institutional Review Board [IRB] of the 37 Military Hospital, Accra (37MH‐IRB/PhD/IPN/817/23). Both deathcare workers and their managers gave verbal and written informed consents to participate after the objective of the study was explained to them. Moreover, the study was performed in accordance with the principles of the Declaration of Helsinki on medical research involving human subjects, the seventh edition [47]. Furthermore, the study was conducted consistent with the Completed Consolidated Criteria for Reporting Qualitative Studies (COREQ). Meanwhile, we employed a Clinical Psychologist to provide psychological support to both the participants and researchers before, during and after the data collection.

## Consent

All authors consented to publish this article.

## Conflicts of Interest

The authors declare no conflicts of interest.

## Supporting information




**Supporting file 1:** puh270205‐sup‐0001‐Appendix.docx

## Data Availability

The authors have nothing to report.
