# Supplementary material for: Lived Experiences of Deathcare Workers in Managing Infectious Dead Bodies
Source: Public Health Chall. 2026 Feb 27;5(1):e70205. doi: 10.1002/puh2.70205 (PMC12947765; doi:10.1002/puh2.70205)
Supplement: Supplementary file 1 — Supporting file 1: puh270205‐sup‐0001‐Appendix.docx [file PUH2-5-e70205-s001.docx]

**Appendix A**

**PARTICIPANT INFORMATION SHEET**

Dear Deathcare Worker

We are researchers from the University of Cape Coast conducting a study titled: “**Lived Experiences of Deathcare Workers in Managing Infectious Dead Bodies**” The aim is to explore the experiences of deathcare workers who manage infectious dead bodies and the psychosocial hazards associated with their work, at selected deathcare facilities in the Central and Western North regions of Ghana. We are contacting you because we trust that as a deathcare worker you have a rich experience that would help shape the outcome of this study. Note that in line with the study aim, we may develop a conference paper or publish an article from the findings. Be assured that no portion of our final report will expose your true identify or your organisation. The interview is estimated to take about 30–45mns of your time. Be further assured that your responses will be treated with the utmost confidentiality, and therefore your honest and complete responses is hereby requested. You are free to withdraw from the study at any stage if you do not wish to continue, even after consenting to participate. We also wish to inform you that the study poses no harm to you or your organisation.

Therefore, if you agree to be part of this research, please sign/thumb print in the space provided below.

Signature/thumb print: ………………………

Date: ……………………

For any further information, please contact me Botha, Nkosi Nkosi on +233242766087, Drs Edward Wilson Ansah on +233247703379 and Cynthia Esinam Segbedzi on +233244974525. Thank you in advance for your cooperation.

**INTERVIEW GUIDE FOR DEATHCARE WORKERS**

**Part I**

Demographic characteristics:

Age: ………………..

Sex: ……………………..

Level of formal education: ……………..

Years of experience: …………………..

**Part II**

**1. How do you manage Infectious Dead Bodies (SARS-CoV-2, Cholera, etc.)?**

Prompts:

*i. How were you prepared to handle infectious bodies?*

*ii. What measures are in place to protect you against infectious diseases?*

**2. What safety hazards do you experience during work?**

Prompts:

*i. What psychosocial safety hazards do you experience during work?*

**3. Is there any additional information you would like to provide?**

**INTERVIEW GUIDE FOR DEATHCARE MANAGERS**

**Part I**

Demographic characteristics:

Designation: …………………..

Years of experience: ……………

Gender: …………………..

**Part II**

**1. How does your facility manage Infectious Dead Bodies (SARS-CoV-2, Cholera, etc.)?**

Prompts:

*i. How do you prepare your deathcare workers to handle infectious bodies?*

*ii. What measures do you have in place to protect them from infectious disease?*

**2. Is there any additional information you would like to provide?**

Thank you in advance for your cooperation.

| **OBSERVATION CHECKLIST I** | | | | | | | | | |
| --- | --- | --- | --- | --- | --- | --- | --- | --- | --- |
| **ON-SITE SAFETY PRACTICES AND USE OF PERSONAL PROTECTION EQUIPMENT** | | | | | | | | | |
| **On-site safety practices** | | | |  | | | | | |
| **Practices** | | | | **Observations** | | | | | |
| Hand/personal hygiene | | | |  | | | | | |
| Handling of dead bodies | | | |  | | | | | |
| Availability and use of PPE | | | |  | | | | | |
| **OBSERVATION CHECKLIST II** | | | | | | | | | |
| **WORKING ENVIRONMENT AND OTHER SAFETY REQUIREMENTS** | | | | | | | | | |
| **Some essential safety features** | | | | | | | | | |
| **Feature** | | **Description** | | | | | | | **Observations** |
| Floor | | Hard and durable | | | | | | |  |
|  |  | Moisture resistant and easily cleaned | | | | | | |  |
|  |  | Floor ducts and trenches absent | | | | | | |  |
|  |  | Junction between walls and floors well sealed | | | | | | |  |
| Walls | | Thick, durable and permanent | | | | | | |  |
|  |  | Fitted with pale blue colour tiles up to the  ceiling | | | | | | |  |
| Doors | | Wide sliding and fly proof | | | | | | |  |
| Corridors | | Wide (not less than 8ft) | | | | | | |  |
| Water  supply | | Regular (hot and cold) | | | | | | |  |
|  |  | Adequate number of sinks | | | | | | |  |
|  |  | All taps within working area elbow operated | | | | | | |  |
| **Other safety requirements** | | | | | | | | | |
| **Item** | | | **Available** | | | **Functional** | | **Remarks** | |
|  |  |  | **Yes** | | **No** | **Yes** | **No** |  |  |
| Air condition | | |  | |  |  |  |  | |
| Fire extinguishers | | |  | |  |  |  |  | |
| Electrical  wires/fittings | | |  | |  |  |  |  | |
| Waste bins | Black | |  | |  |  |  |  | |
|  | Brown | |  | |  |  |  |  | |
|  | Yellow | |  | |  |  |  |  | |
| Lumination | | |  | |  |  |  |  | |
| Rest room | | |  | |  |  |  |  | |
| Bathroom | | |  | |  |  |  |  | |
